# Lipid Profiling in Cancer Diagnosis with Hand-Held Ambient Mass Spectrometry Probes: Addressing the Late-Stage Performance Concerns

**DOI:** 10.3390/metabo11100660

**Published:** 2021-09-28

**Authors:** Lauren Katz, Alessandra Tata, Michael Woolman, Arash Zarrine-Afsar

**Affiliations:** 1 Department of Medical Biophysics, University of Toronto, 101 College Street, Toronto, ON M5G 1L7, Canada; lauren.kaufman@mail.utoronto.ca (L.K.); michael.woolman@rmp.uhn.ca (M.W.); 2 Techna Institute for the Advancement of Technology for Health, University Health Network, 100 College Street, Toronto, ON M5G 1P5, Canada; 3 Laboratorio di Chimica Sperimentale, Istituto Zooprofilattico delle Venezie, Viale Fiume 78, 36100 Vicenza, Italy; ATata@izsvenezie.it; 4 Department of Surgery, University of Toronto, 149 College Street, Toronto, ON M5T 1P5, Canada; 5 Keenan Research Center for Biomedical Science & the Li Ka Shing Knowledge Institute, St. Michael’s Hospital, 30 Bond Street, Toronto, ON M5B 1W8, Canada

**Keywords:** ambient mass spectrometry, untargeted lipidomics, untargeted metabolomics, lipid profiling, cancer diagnosis with ambient mass spectrometry

## Abstract

Untargeted lipid fingerprinting with hand-held ambient mass spectrometry (MS) probes without chromatographic separation has shown promise in the rapid characterization of cancers. As human cancers present significant molecular heterogeneities, careful molecular modeling and data validation strategies are required to minimize late-stage performance variations of these models across a large population. This review utilizes parallels from the pitfalls of conventional protein biomarkers in reaching bedside utility and provides recommendations for robust modeling as well as validation strategies that could enable the next logical steps in large scale assessment of the utility of ambient MS profiling for cancer diagnosis. Six recommendations are provided that range from careful initial determination of clinical added value to moving beyond just statistical associations to validate lipid involvements in disease processes mechanistically. Further guidelines for careful selection of suitable samples to capture expected and unexpected intragroup variance are provided and discussed in the context of demographic heterogeneities in the lipidome, further influenced by lifestyle factors, diet, and potential intersect with cancer lipid pathways probed in ambient mass spectrometry profiling studies.

## 1. Introduction and Problem Statement

Based on the close relationship between lipid metabolism and cancer formation/progression [1], tissue pathology determinations (cancer versus healthy or differentiation between various types of the same cancer) through lipid profiling with ambient mass spectrometry (MS) has received much traction [2,3,4,5,6,7,8,9,10,11,12,13,14,15,16,17]. A variety of aerosolization and liquid extraction methods [3] (some utilizing handheld sampling probes capable of desorbing tissue molecules under ambient conditions) are coupled to MS analysis after ionization, generating a lipid profile signature from the target tissue specimen. Through comparing the overall mass to charge (*m*/*z*) pattern of this “crude” tissue lipid profile (or signature) generated in the absence of chromatographic separation, to a library of previously collected lipid profile signatures characteristic to various tissue pathologies, rapid identification of said pathologies (cancer, infection, or inflammation) has been made possible within a few seconds of data collection and analysis [4]. The data analysis step in ambient MS often uses multivariate decomposition methods [4] but has also been augmented with machine learning, [18] artificial intelligence [19], and deep learning methods [20] to mine further nuances in tissue classifying molecular profiles. 

Targeted MS approaches for the identification of necrotic tumors using certain ceramides [21] and other unique lipids for select kinase activity monitoring [22] have been reported. While there are additional cases in which a single oncometabolite such as *N*-acetylaspartate (for differentiation of healthy neuronal tissue from glioma) or 2-hydroxyglutarate (for determination of isocitrate dehydrogenase mutation status [8,10,23,24,25,26]) is used in a targeted analysis, many ambient MS studies utilize the overall ionic pattern of tissue lipids in an untargeted analysis or a subset thereof (e.g., most distinguishing ions as from a parsimonious method that uses feature reduction [27]) as the distinguishing “biomarker” [2,3,4,5]. These developments implicitly redefine the “classic” notion of a disease biomarker from a single (often protein) molecule that is reproducibly validated across many specimens in a “targeted” analysis, to a pattern of disease specific (and thus disease classifying) metabolites or lipids analyzed in their unique ionic states in an “untargeted” manner, often acquired across far fewer independent specimens. The new “biomarker”, in other words, is a multidimensional classifier that is represented by its coordinates on a multivariate decomposition plot. The act of disease identification using said classifier thus boils down to tracking the juxtaposition of the multivariate coordinates of a query mass spectrum on the multivariate decomposition plot of the reference signature library. Here, a pre-defined certainty level to call out positive hits (overlapping with the model) from non-overlapping (or negative) hits is used.

**Figure 1:**
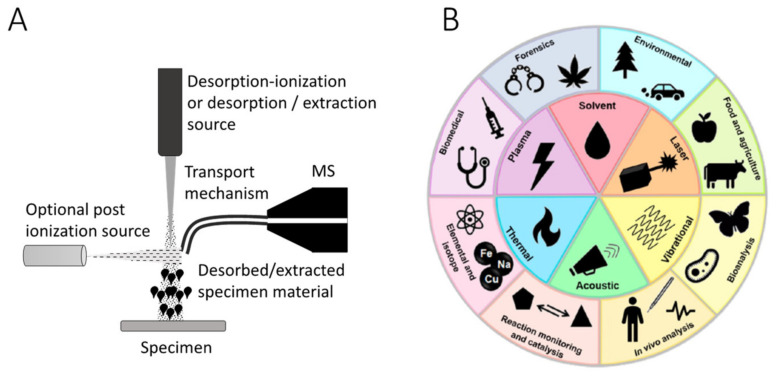
Principles and high-level overview of the mechanisms of ambient mass spectrometry. (**A**) Schematics of a typical ambient MS source. A desorption−ionization source or an extraction source provides desorbed or desorbed and ionized molecules from a specimen under ambient conditions. Inclusion of post ionization is optional and enhances the signal in select cases. A mechanism for the transport of extracted/desorbed materials to the mass analyzer is included that may involve flexible tubing for long (>2 m) transport of surgical aerosols as in iKnife [14], laser desorption plume as in SpiderMass [31] and PIRL-MS [32], or water (as solvent) extracted tissue content as in the MasSpec Pen [17]. The ionization step can take place anywhere between the specimen surface or close to the mass analyzer. A notable difference is lack of chromatographic separation of analytes prior to MS analysis. (**B**) A high-level overview of core ambient desorption/ionization technologies and related applications. Here, a variety of desorption/ionization methods consistent with the schematics provided in panel A are used to generate a multiple application base that ranged from surgical to material to environmental or forensic explorations. Reprinted (adapted) with permission from [3], Copyright 2019 American Chemical Society.

To date, diverse ambient MS methods have been employed to address a wide variety of clinical cases [2,3,4,5]. Overall, very good analytical performance metrics for lipid or metabolite pattern matching in ex vivo or in situ tissue explorations across tens to hundreds of independent clinical specimens have been reported [2,3,4,5]. Often, by simply splitting the acquired (albeit highly limited) datasets into training (model building) and test (recognition) subsets, predictive multivariate classification models are created and validated based on statistical association with the disease state signal. The success of ambient MS profiling in the context of such limited analytic validation steps has largely been hailed as resulting in promising new avenues for rapid and accurate cancer diagnosis. Beyond lipidomic analysis of solid tissues with untargeted ambient MS methods described above, utility of plasma lipidomics [28] for cancer diagnosis is also gaining momentum [29]. A detailed review of technologies and various applications of ambient MS sources (beyond the scope of this manuscript) is provided elsewhere [2,3,4,5]. However, in summary, an ambient MS analysis source often employs an extraction mechanism, desorption−ionization, or a desorption followed by post ionization wherein the specimens are subjected to extraction of molecular content largely in the absence of extensive pre-processing and under ambient conditions (Figure 1A). A variety of extraction or desorption methods are used, leading to a diverse set of applications ranging from clinical diagnosis to food safety and pharmaceutical explorations (Figure 1B). Laser-based ambient ion sources have been extensively reviewed recently by our group [30], and need not be repeated here. It must, however, be emphasized that as of late, the majority of hand-held ambient MS probes (focus of this manuscript) do not utilize pre-separation of analytes (e.g., by means of chromatographic or ion mobility). As such, direct infusion (as done in conventional lipidomics) best describes the state of the art in ambient MS methods. This poses a fundamental challenge in the applicability of analysis tools such as Compound ID and content look up databases such as LipidMaps to ambient MS data. 

**Figure 2:**
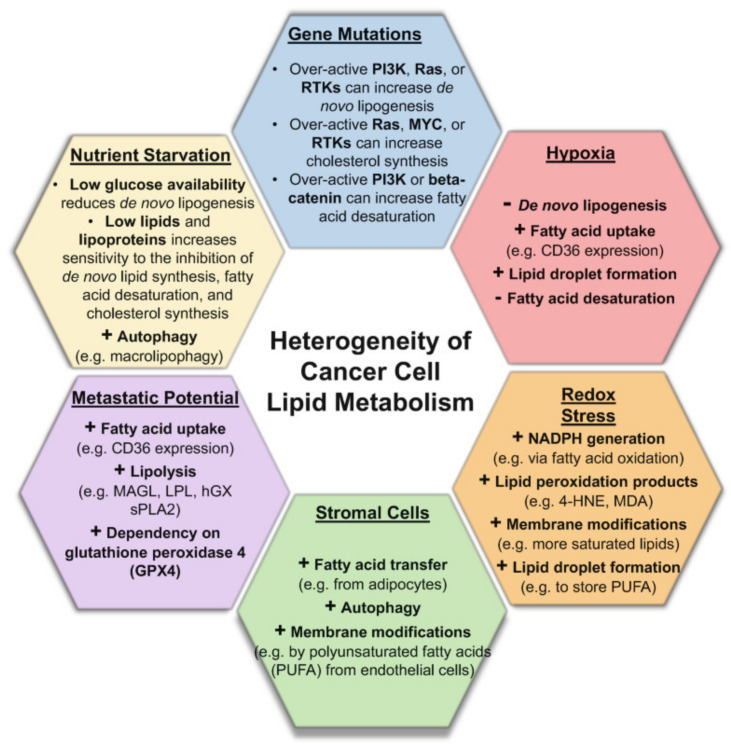
Heterogeneity of lipid metabolism in cancer. Overview of multiple factors that can influence lipid metabolism in cells, creating additional molecular heterogeneities that must be captured in ambient MS profiling studies through use of a large, balanced, and heterogenous sample set. These factors can be external (diet) and can arise due to intrinsic biological architecture (stroma), mutations, and the tumor microenvironment (hypoxia). Reproduced with permission from [43].

By the same token that a single disease biomarker molecule must pass rigorous analytical, regulatory, and late-stage performance surveillance (population validation) to ensure high sensitivity and specificity, lipid and metabolic profiling with ambient MS must also be subjected to the scrutiny of the same guidelines and principles. In this quest, lessons can be learned from the failure of many protein biomarkers to reach the clinic [33,34,35], and these lessons can be applied to the nascent field of rapid tissue pathology through lipid profiling with ambient MS. Five important differences set lipid profiling with hand-held ambient MS probes apart from many single molecule (protein) biomarker discovery and validation efforts. First, the majority of published ambient MS studies used a small, and rather homogenous population of clinical specimens in the analytic validation step to assess the accuracy, specificity, and sensitivity of the tissue pathology predictions [36]. While data analysis methods have been introduced to boost predictive power of limited models using “cumulative learning” approaches with some success [37], the issue of a model’s poor predictive ability, arising from small size, deserves special attention. Second, tissue lipid profiles are likely to be influenced by intrinsic population genetic heterogeneity, diet [38,39,40], and other tumor microenvironment factors such as stromal content and presence of hypoxia, among others (Figure 2). It has been shown that changes in diet and lifestyle interventions alter tissue lipidomes in mice, but not all tissues are affected to the same extent or show the same altered lipidomic pattern [41]. Third, our collective understanding of the underlying lipid metabolic pathway heterogeneities [42] remain limited, as few studies have investigated large-scale variation of lipidome across healthy populations. Fourth, most published ambient MS reports fail to further determine the molecular identities of important tissue classifying lipid molecules. This makes utilization of metabolic pathway knowledge in a rational approach to further refine them to those least influenced by population heterogeneity and diet a daunting task. Fifth, by definition, tissue lipid profiling is an “untargeted” MS approach that, in the absence of chromatographic or ion mobility separation, is vulnerable to population noise perhaps to a greater extent than a single known and well characterized molecule in a targeted approach would be. While parsimonious analysis methods that use feature reduction may be less susceptible to population noise compared with wide mass range models, discovering a mechanistic link between biomarker and disease state further buttresses biomarker validation beyond just what is inferred from the strength of its statistical association with disease state. Statistical association as the sole criteria for biomarker identification and validation in an untargeted approach can be misleading if not supported by additional mechanistic verification [35]. The unequivocal identification of metabolites must utilize high resolution mass spectrometry, tandem MS analysis for diagnostic fragment identification in conjunction with additional separation (ion mobility and/or chromatographic) methods as an orthogonal approach. 

This review provides a critical assessment of the dangers associated with the use of small initial (and often unbalanced) sample sets [44,45,46,47] in pattern recognition [36] and lack of late stage validation with respect to the utility of the discovered lipid pattern for a high sensitivity and specificity performance across a diverse population [36]. We then accordingly provide recommendations drawn from the growing field of untargeted MS analysis in food sciences facing similar challenges in validation strategies [48,49,50,51]. To detect an unlimited set of potential adulterants with an untargeted approach, a large and diverse population of authentic products must be examined [52]. We first review broad stroke principles of analytical, late stage, and regulatory validations, and then attempt to extend the implementation of predictive markers from early phase trials [53] to large scale validation [54], drawing further upon sources of failures in the protein biomarker discovery world [33,34,35,55,56] to provide validation strategies for ambient MS profiling that are most suitable to minimize late stage performance concerns across diverse populations.

## 2. State of the Art

Any method intended for clinical decision making or use at the bedside must offer, over a pre-defined intended use, a precise, accurate, robust, sensitive, and specific performance within a reportable range that is reproducible across many sites, users, specimens, and is further uninfluenced by instrumental and environmental factors and duty cycle (carry over), and should additionally meet specific safety and use site compliance requirements [57,58]. These constitute analytic performance matrices that have recently been reviewed in the context of ambient MS method use at the bedside [57]. When laboratory developed tests (LDTs) [59] are used to develop a new targeted MS approach, often, a homogenous, relatively small sample set sourced from patients local to the laboratory site or region may be used as the first step for validation. This strategy, however, leaves the analytic validation step vulnerable to population variation, leading to late-stage performance concerns not addressed during first trials. While the pharmaceutical industry rigorously engages in post-marketing surveillance campaigns to monitor the performance of novel therapeutics (in terms of safety and also efficacy), LDTs, if not validated using a sufficiently diverse (or statistically reduced or demographically matched) test case, remain very vulnerable to population level poor performance [60], as seen in a number of failed biomarker cases that showed good performance in the laboratory but failed to perform well across the population in the absence of demographic matching [54,55,56]. Here, limited information such as changes in a marker in response to disease progression, pharmacological intervention, and crosstalk therein, will further complicate the co-efficient of similarity-based methods in the absence of careful demographic matching with the model. While biomarker driven targeted approaches (or those with statistically reduced datasets) that rely on identified disease specific peaks may be somewhat shielded against such variations, similarity coefficient-based comparison methods that rank a query spectrum against the spectra from a collection of diseases must be more closely scrutinized. Currently, two untargeted MS methods for pathogen strain identification have broken through the regulatory barrier [61,62]. Building on a possible pathway of LDTs [59], the USP Pharmacopeial Convention provides guidelines for analytic validation of untargeted mass spectrometry methods in food sciences, quality control, and for the detection of adulteration [49,50,51,63]. Further resources for bioanalytical method validation are available through the FDA [64], and a recent adaptation of pharmacopeial and bioanalytical methods validation strategies specifically tailored to ambient MS methods has been recently published [57] in an attempt to standardize an analytic validation workflow for this technique in cancer research. This review (by our group) attempted to put forward guidelines for rigorous analytic validation, including suitable sample sizes [65] according to power calculation [66], as recommended for metabolic phenotyping [44,45] and stayed faithful to the minimum reporting standards for chemical analysis [46,67]. However, late-stage validation concerns for rapid tissue lipid profiling that use lessons learned from failures of targeted single molecule protein biomarkers to reach the clinic are not reviewed in depth, and thus form the basis of this report. Moreover, a previous report by our group qualitatively addressed the influence of tissue molecular heterogeneity on ambient MS profiles in the context of choosing the correct disease model [68]. This review further details the origins of this heterogeneity and provides recommendations for optimal design of validation studies to shield against the potential confounding effect of population heterogeneity on ambient MS profiles. 

### 2.1. Molecular Heterogeneities in Biological Tissues Impact Metabolome and Lipidome Profiles

As discussed in the section above, lipids have been shown to play diverse roles in many cellular functions. It was postulated (a decade ago) that lipidomics would evolve to play a critical role in expanding our understanding of disease states [69]. As speculated then, lipidomic technologies have indeed matured considerably to broaden our understanding of how lipid metabolism correlates with disease biology [70], especially in cancer, where lipids [71,72], in addition to being of diagnostic value [29], may be involved in a number of regulatory pathways [43,73], thus showing additional promise as therapeutic targets [74,75]. While our ability to effectively target cancer through modulating lipid metabolism is still an open question [76], there is convincing evidence that there exists a significant amount of molecular heterogeneity in cancer cells [77,78]. The exact ramification of this molecular heterogeneity in terms of divergent metabolic (or lipidomic) pathways is only beginning to emerge in select cases [43,78,79,80,81,82,83,84,85,86,87], but there is convincing evidence that many factors, including adaptive evolution (to treatment [88]), microenvironment components (stroma and inflammatory cells), and hypoxia, among others, can influence cancer cell metabolism [89], and in some cases directly affect lipidomic profiles [43,81,84,90,91,92]. Of notable importance is a study of lipid metabolism in breast cancer that suggests a differential role of essential and non-essential lipids in metabolic profiles of inflammatory and cancer cells within the tumour tissue [91]. In the absence of spatially resolved ambient MS analysis (or single cell lipidomics [93]), to better capture these heterogeneities, alongside those arising from metabolic adaptability of cells to their environment [94], at the very least, a large population base must be included in the analysis, particularly in the initial model building stage. The issue of spatial resolution (often not at single cell levels in prominent ambient MS methods) causes sensitivity of signal to tumour cellularity, as shown in a DESI-MS lipidomic profiling study of brain cancers [95] and the localization of high 2-hydroxyglutarate (2HG) signal levels in the regions of glioblastoma tumours with a dense cellularity [10]. While this sensitivity can be utilized to estimate tumour cell percentage [96], which is in its own right a useful clinical indicator currently only obtainable by time consuming histologic staining and microscopy, caution must be exercised in the interpretation of ambient MS profiles associated with residual disease presence. Residual HER2 expression within breast tumours [97] in a proteomic MALDI-MS study has been shown to influence receptor status predictions [98].

In a recent study aimed at creating predictive molecular models of breast cancer receptor status with ambient DESI-MS [99], intrinsic molecular heterogeneity in HER2 receptor patients [100] resulted in dramatically reduced accuracy of DESI-MS models for the determination of this receptor status compared to that of the estrogen (ER) and progesterone (PR) receptors from DESI-MS models. In a similar vein, in a previous ambient MS profiling study of pediatric medulloblastoma cancer molecular subgroups, an aberrantly high content of stroma or an infiltration of healthy tissue in the specimens to be classified, not accounted for in the model, led to misclassification or failed classification [9]. This study highlights the significance of comprehensive molecular models that capture the expected molecular heterogeneity associated with the infiltration and microenvironment. Further emphasizing the impact of the architectural (and hence molecular) heterogeneity of cancer, DESI-MS modeling of ovarian high-grade serous carcinoma and serous borderline ovarian tumours resulted in misclassification of a few specimens that contained microcapillary growth patterns and other architectural complexities common to invasive carcinoma features associated with a specimens’ unique pathology [101]. 

In addition to these discoveries that highlight the extent of the influence of tissue molecular heterogeneity on the predictive power of MS-based modeling, it has long been recognized that diet may modulate both plasma and certain tumour lipids [102]. Diet exchange between demographic groups has been shown to alter the metabolome (and cancer risk [103]), and chronic exposure may mark an effect on the metabolome [104]. Cancer cells are known to scavenge nutrients from their environment, especially under aggressive growth or inadequate perfusion [105], creating an additional link between diet and metabolism. Furthermore, the availability of precursor lipids diffused to the tumour site [106] is shown to create additional spatial heterogeneities in tissue metabolic states in the vicinity of cancer [107]. A high fat diet has been shown to alter lipid metabolism in cancer and influence the interplay between normal and cancer cells [108]. This relationship is exemplified elegantly by the crosstalk between adipose tissue (storage for dietary lipids [109]) and tumors [110], with cancer-associated adipocytes [111] providing adipose-derived lipids for cancer progression [112] and proliferation [113], with stipulated therapeutic potentials [114,115]. 

Further complicating the interplay of diet, adipocytes, and lipid uptake in cancer metabolism discussed above, alcohol consumption is shown to perturb adipocytes, promoting lipolysis [116,117,118] and thus potentially influencing cancer cell metabolism by modulating released precursor lipids. In addition, there has been a further recognition of the influence of gender and age on the composition of the plasma lipidome [119,120,121]. While the influence of plasma lipidome variations on solid tumour profiles is yet to be systematically investigated, a picture thus emerges that to improve the diagnostic accuracy of lipid profiling for cancer characterization with ambient mass spectrometry, diet, gender, and lifestyle factors, among others (e.g., clinical history regarding treatments received) must be taken into consideration, and that ideally, spatially resolved analysis is required to capture intratumoural lipidomic or metabolomic heterogeneities. While the latter is not possible or technically feasible, significantly large sample numbers to capture as much of the population level or batch effect variances [57] will be helpful.

### 2.2. Initial Statistical Modeling Should Be Based on Sufficient Sample Numbers

**Figure 3:**
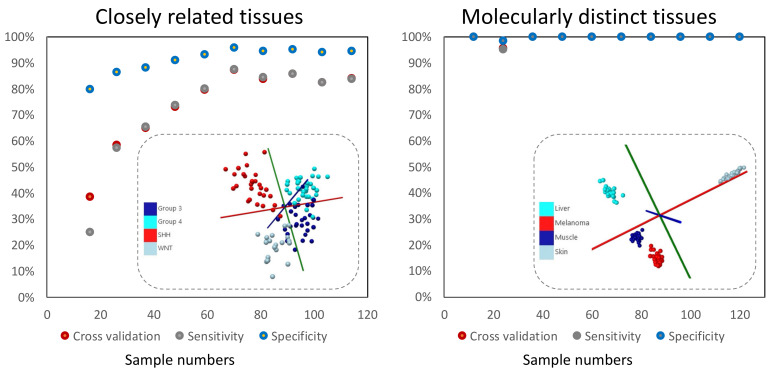
The influence of sample size on the predictive power of ambient MS methods. In this figure we re-analyzed previously published [9] and newly acquired picosecond infrared laser mass spectrometry (PIRL-MS) data from two cases of 4 closely related tissues (subgroups of human pediatric brain cancer medulloblastoma) and 4 distinct tissue types (murine melanoma and additional liver, muscle and skin tissues). Each dataset is comprised of a 10-s ambient PIRL-MS sampling (*m*/*z* range 100–1000 Da) subjected to principal component analysis and linear discriminant analysis (PCA-LDA), as described previously [9]. Cross validation was performed on AMX [144] using a 20% leave out [9], and was used to calculate the specificity and sensitivity. For each specimen, one 10-s PIRL-MS spectrum has been included. However, in keeping with the recommendations of Nyangoma et al. [135] (see above), multiple sampling of each specimen towards developing the extent of intrasample variation has been reported in our original publication [9]. As can be seen here, where there is significant intrasample variability (large spread of data points for each class in the PCA-LDA scores plot) and where classes are more molecularly alike, larger sample numbers are required to reach prediction power plateau compared with cases where less variant, molecularly distinct classes are compared. In this comparison, each class contained ~30 specimens (and one PIRL-MS spectrum per specimen).

The impact on lipidomic profiles of tissue molecular heterogeneity discussed above further supports the much needed emphasis on suitable sample size to produce significant results in pattern recognition [36,122,123] and related efforts [36], as well as in multivariate and high dimensionality data analysis [36,44,45,47]. Effective sample numbers that result in statistically significant profile models must be optimized for each case study and incorporated in the earliest phase of ambient MS profile model building. Here, inter- and intra-group variance in lipid profiling data is a key determinant of the overall sample sizes required to differentiate between closely related or molecularly distinct tissue types, especially if elevated levels of intra-group heterogeneity and variance are present in the dataset. In Figure 3, we illustrate this point by re-analyzing published data [9] corresponding to the principal component analysis and linear discriminant analysis (PCA-LDA) of 4-component groups, closely related and molecularly distinct. As illustrated in this figure, larger sample numbers per group are required to effectively distinguish closely related groups compared to molecularly distinct groups. Here, analysis of so-called “learning curves” in the related domain of micro-array analysis [124,125] have shown promise in determining effective sample sizes [126], and a minimum of 75 specimens per class have been suggested [127]. Here, there are parallels to draw from proteomics using Matrix Assisted Laser Desorption Ionization Mass Spectrometry (MALDI-MS) profiling [128,129,130,131], where guidelines for determining the appropriate sample size for clinical proteomic profiling studies have been put forward, using a linear mixed model that allows for the inclusion of estimates of biological and technical replicate variance in a given experiment [132]. In a similar vein, by extending previously published approaches [133,134], the effect of biological variance (e.g., intratumoural heterogeneity), as an important determinant of the overall sample size required, has been examined towards a method that allows for the adjustment of expected biological variance in calculations of sample size [135]. A central tenet of this approach is a rigorous replicate analysis (i.e., multiple sampling) of each specimen towards determination of the intraclass heterogeneity. Here, averaging replicates and using this value in class differentiations is discouraged as averaging results in loss of valuable information regarding intrasample variability [135]. More interestingly, beyond basic multivariate methods, such as linear discriminant analysis (LDA) [136], least absolute shrinkage and selection operator (LASSO) [137], machine learning approaches such as support vector machine (SVM) [138], and random forest (RF) [139], which require extensive pre-processing of mass spectral data, convolutional neuronal networks have been proposed to offer a higher accuracy of prediction without the need for data pre-processing [140,141]. The predictive power of these methods, however, sharply decreases with small sample sizes [142,143]. While cumulative learning with convolutional neural networks have been proposed to utilize smaller mass spectrometry datasets [37]; the future augmentation of ambient MS data analysis methods with artificial intelligence is bound to make a larger demand on diversity of training datasets and careful considerations of appropriate sample sizes as discussed above.

### 2.3. Lessons Learned from Metabolomic and Proteomic Biomarker Discovery and Food Sciences

The horizontal growth of untargeted ambient mass spectrometry profiling has taken place in the absence of an effective dialogue with researchers in the areas of metabolomics or proteomic biomarker discovery. While most of the “conventional” biomarker discovery studies use targeted approaches, there are challenges in metabolic profiling study design (reported previously [145,146]) that may be applicable to the untargeted ambient MS analysis of tissue molecular profiles. It must be emphasized that a large fraction of true biomarker discoveries (those that have been validated in controlled laboratory or through early-stage discovery or small population studies) fail to break through the clinic [33,35,55]. While the sources of such failures can include many factors such as low added value to prognostic or clinical utility (stemming from improperly defining the clinical need) [33], underestimating cancer heterogeneity (extreme case selection) among others reviewed elsewhere [55,56], a low sensitivity or specificity across the wider population [54] in late-stage or multisite trials have also been reported [55]. As an example, B7-H4, a novel membrane bound protein proposed as a marker for ovarian cancer [147], failed due to the large variance seen across multisite validation efforts [148]. Further buttressing the importance of careful study design, lysophosphatidic acid (LPA) [149] failed to become a reliable marker for gynecological cancers as the debut publication used a non-standard sample processing protocol [150]; thiosulfate failed to stand the scrutiny of additional benchmarking against other orthogonally validated markers for prostate cancer despite early stage promise [151]. Ambient MS profiling is not immune to the hurdles and challenges reviewed elsewhere [33,55,56], especially those that highlight the importance of large study validation using a diverse population [54,55], reducing the hype based on limited initial findings [55]. Extrapolating parallel sources of failures seen in these fields [54,55,56] to the design of ambient MS profiling studies will be helpful. Table 1 summarizes a few key points of value for consideration by investigators using ambient MS profiling in their research. In essence, biomarker discovery efforts may face pitfalls in all stages of discovery, validation, translation, evaluation, and implementation. As illustrated in Table 1, small studies or those that use extreme cases without careful validation and use of replicates, rationalization of discordant results, or careful design of a randomized trial across sufficiently diverse set of parameters are likely to fail. In a similar vein, a poorly defined clinical case that lacks clear added value to the clinical practice is unlikely to pass the evaluation or implementation stage. Lastly, solutions that pass the scrutiny of the above are not necessarily bound to succeed either. A solution that passes implementation must improve outcomes or offer performance added value across diverse settings and must also not be cumbersome to implement (be easy to use). Here it must be emphasized that properly defining the key parameters listed in Table 1, such as “diverse setting”, “sufficiently large datasets”, “ease of use”, and “clear added value” is not an easy undertaking. Clearly defining these factors requires an intimate understanding of the underlying diversity in the molecular make up of target molecule(s) across a large number of factors, a feat that is far more complicated in untargeted analyses. Here, the untargeted metabolomic analysis of adulterated food provides helpful parallels, especially in revealing the importance of capturing heterogeneity in the initial statistical model studies. Due to the untargeted nature of the validation protocols, either a vast number of potential adulterants must be included in the initial model building efforts, or a large variety of authentic products from diverse origins must be studied and their molecular fingerprints catalogued as authentic [49]. In food authentication, the US Pharmacopeia guidelines explicitly note that samples in the reference set must fully encompass all of the variability of the food product under study [52]. Large sets of samples enable the appropriate inclusion of biological variance, and further including replicates is encouraged to minimize unexpected variations [49,51]. This scenario closely parallels lipid profiling studies of highly heterogenous cancers wherein as much of the existing inter- and intra-specimen heterogeneities as possible must be captured. Further emphasizing the importance of comprehensive reference datasets that capture the entire range of expected specimen heterogeneity, in an evaluation of certified reference materials used for oregano authentication with untargeted ambient mass spectrometry, a false positive was identified; the model failed to classify a certified sample from South America as authentic due to the training set utilized having been only populated with certified oregano specimens with a European origin [152]. Similarly, two newly established untargeted molecular models for extra virgin olive oil authentication with gas-chromatography fingerprinting failed to classify aged samples from a previous harvest season [153]. The limitation of insufficient heterogeneity in the reference dataset was overcome by focusing on the presence of adulterant sunflower oil instead, in order to assess olive oil samples from new geographical regions not included in the reference model [154]. This strategy was suitable as there was only one type of known adulterant expected, and may not be applicable to all ambient MS profiling studies. Here, a comprehensive review of the challenges associated with untargeted fingerprinting methods used in food authentication has been published that emphasizes frequently updating the model and revising them as new data become available [155]. This puts forward a new vision for the creation of intelligent, self-improving molecular models for cancer diagnosis with untargeted ambient MS lipid profiling wherein the availability of new data post analytic phase (implementation) can result in naturally evolving models that capture additional heterogeneity from diverse sites, and across varied socio-demographic strata. A key to the successful implementation of this vision, besides artificial intelligence models, is a rigorous definition of ground truth (i.e., pathology) to ensure only spectra from validated authentic samples are included in the revised dataset. 

### 2.4. Moving beyond Statistical Associations 

A caveat of supervised untargeted ambient MS analysis is its strong dependence on statistical correlation of mass spectra with ground truth information (e.g., pathology). The untargeted nature of lipid profiling that often utilizes many features (*m*/*z* values) creates additional vulnerability to population level variations. While restricting the mass range to smaller regions may provide some benefit, the analytic validation guidelines summarized above (and discussed previously [57]) do not per se call for identification of tissue classifying lipids. Therefore, establishing a further mechanistic link between lipid profiles and disease state is not always performed. When small sample sizes pose a challenge on study significance [36], boosting confidence in association of lipidomic patterns with the disease state uncovered in the validation phase through a mechanistic link will be advantageous [156]. Furthermore, as our knowledge of the metabolic pathways [42] advances [157,158], controlled xenograft models continue to provide an additional test-bed for orthogonal validation of certain lipids involved in metabolic pathways [159,160,161,162,163], containing selective mutations in lipid synthesis machinery [22]. Feature reduction to perform disease classifications with as few tissue-specific and highly distinguishing lipids as possible (through low complexity or sparse analyses) [9] is an attractive strategy to further shield against population noise that may alter the spectra without influencing all the strongly classifying peaks. This rational approach further allows for the inspection of potential crosstalk between dietary pathways (food metabolome [164]), and identified lipid marker(s) pathways to anticipate heterogeneities (utilizing human metabolome database insights [165,166,167]) or to iteratively reduce *m*/*z* features towards development of an initial model that is robust in terms of tissue classification and uninfluenced by population level noise or heterogeneity. An example of this is an investigation of molecular signatures of ischemic heart tissue (myocardial infraction) using spatially resolved ambient MS profiling with machine learning mining and molecular identity determinations, wherein a role for taurines in the infarction process was discovered [168], consistent with the physiological role of taurines in myocardial tissues [169]. A growing body of evidence suggests that diet-induced depletion of taurines influences cardiomyopathy and its supplementation is helpful in attenuating the degradation noted [170,171]. Rational investigation of pathways in the context of molecular identification could thus prove beneficial in revealing potential crosstalk with diet. Here, an understanding of the basal level variation across healthy population will be an important step in rationally creating suitable molecular models that capture a sufficient level of heterogeneity for the purpose of accurate predictions. 

In addition to providing further support for involvement of a particular metabolite (detected with ambient MS) in a disease process, metabolic pathway information can also be utilized to enhance classification by invoking and subsequently involving additional metabolites, rationally determined to be influenced by said disease pathway, in the classification process. An example of this is reported in a recent work from the Zare group, where rational inclusion of cognate Krebs cycle metabolites, also involved in other cancers [172], in the classification algorithm not only validated the initial set of discovered metabolites relevant for basal cell carcinoma detection, but also improved the classification accuracy of the model used [137]. 

## 3. Recommendations and the Proposed Workflow for Ambient MS Method Validation for Rapid Pathology Determination 

**Figure 4:**
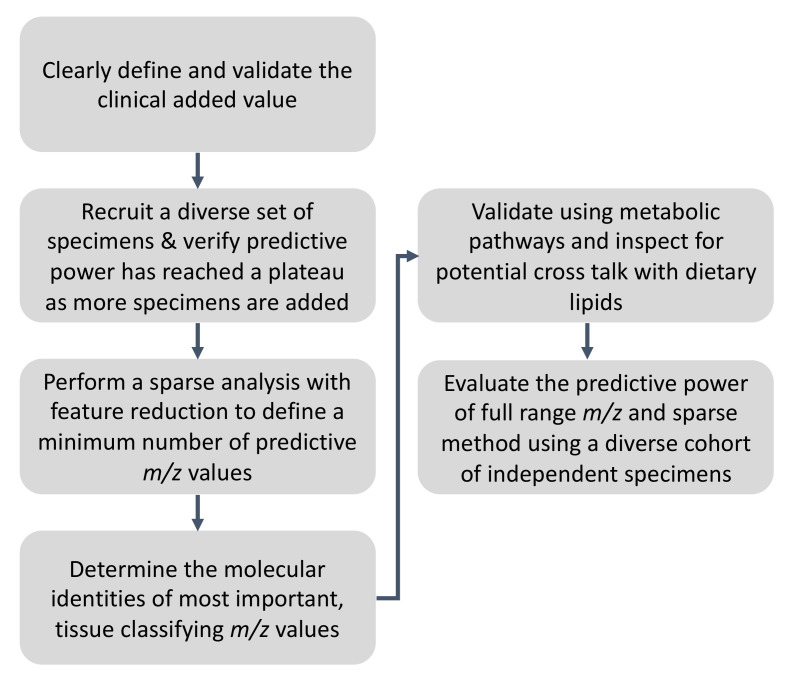
Recommendations for ambient MS profiling workflow focused on utility for rapid pathology determination. Summarizing the material gathered and discussed in this review and major sources of failures in conventional biomarker discovery efforts (Table 1), we have made a high level workflow for ambient MS analysis. This emphasizes proper definition of a useful clinical case, use of diverse sample set that takes into consideration intraclass heterogeneity using rational statistical sample size calculation methods discussed in the text, alongside an impetus to go beyond statistical associations to identify tissue classifying molecules and rationally justify their association using common disease pathway knowledge. This has the added bonus of feature reduction to reduce crosstalk with obvious metabolic heterogeneity factors such as diet. These recommendations are only high-level and should be used in conjunction with lessons learned from Table 1, and material detailed in the text, especially with respect to recommended sample size calculation methods. Providing a one-size-fits-all recommendation for a suitable sample size calculator is beyond the scope of this paper; sample sizes must be optimized for each study.

Based on the points discussed above, and taking an exemplary application of rapid pathology readout, in Figure 4, we propose a workflow for ambient MS analysis of biological tissues. Some of the elements in the proposed workflow may have to be further defined in conjunction with notes of caution and solutions provided in Table 1. We nevertheless propose to, in a general and high-level sense: (1) Define the clinical need (or added value) by performing thorough key opinion leader interviews at the earliest stage possible. For example, non-subjective tissue pathology information from ambient MS is useful. However, whether current standard of care methods have any shortcomings in terms of speed, accuracy, or scope of available information to be delivered by ambient MS must be established first. Beware of the fact that clinical decision making in the standard of care workflow is based on the scope of information currently obtainable. While rewriting the standard of care around new information (now available) is possible, strong justifications (and possibly parallel outcome data) are required to suggest revisions to the standard of care workflows. Rapid diagnosis will not always drastically improve clinical decision making. (2) Use a diverse and balanced sample set at the first possible (initial) modelling effort opportunity. This will allow for the sensitivity and specificity (predictive value) to be established using a heterogenous set early on. Verify how predictive power changes as a function of sample numbers included in the model and increase the sample numbers until the predictive power has reached a plateau (in a “predictive power” versus “sample number”correlation plot). If possible, establish population noise level by including a cohort of healthy specimens in the study and pay special attention to intraspecimen heterogeneity by performing multiple samplings across each specimen. Likewise, perform blind test validation with a diverse set, ideally sourced from a different demographic origin (see Table 1 for pitfalls and redocumentations). (3) Perform a low complexity (or sparse) analyses, and feature reduction to define the minimum number of lipids that can perform the classification without drastically sacrificing predictive power, sensitivity, and specificity. (4) Perform identification of most distinguishing (or tissue-classifying) lipids using orthogonal tandem MS or high resolution, chromatography-enabled MS analysis in conjunction with tools and platforms created for conventional lipidomics or metabolomics such as LipidMaps [173]. HPLC-MS/MS analysis can utilize “pathology guided” tissue section sampling methods such as those with laser capture microdissection. It must be noted that a rational association between markers and dysregulated disease pathways will further enhance feature reduction. Here, correct annotation of identified lipid markers is of exceptional importance also for augmenting the current knowledge of disease pathways and their functional decoding. Unfortunately, inaccurate annotation is common in many lipidomics studies [173,174]. This has created a need for standardization [175] where the use of latest guidelines [173] is recommended. (5) Create a mechanistic link between identified lipids and disease biology if feasible (and validate using engineered xenograft models) and inspect metabolic pathways for possible crosstalk with dietary lipids, performing additional feature reductions towards a set of lipids that provide robust predictive value, specificity, and sensitivity with less expected crosstalk with diet. (6) Evaluate the predictive model of full spectrum and feature reduced models using the validation set and use the model that provides best performance and use this set for further multi-site, multi-user validation by exchanging standardizing and following devised protocols interpretable by multiple users at different sites. Lastly, we highly encourage investigators to define their own user skill set requirements and establish early on who will be implementing the solution; do they possess the right skillsets or find the solution difficult to implement? As stated above, a combination of Table 1 notes of caution and the high-level summary provided in Figure 4 must be considered to address the late-stage performance discordance in adopting an untargeted ambient MS method for bedside use. 

## 4. Conclusions and Caveats

This review paper aims to encourage a dialogue between investigators that use MS profiling for cancer detection and those involved in clinical biomarker validation on the population level. Using a limited initial sample set and validating it with a homogenous specimen set that likely fails to capture the large-scale variance expected at the population level constitutes an important misstep that has led to the failure of many protein biomarkers to reach the clinic. We provided six recommendations to encourage robust and early careful study design that takes into considerations pitfalls of promising protein biomarkers that failed to reach utility at the bedside, to facilitate clinical translation of rapid lipid profiling for accelerated cancer diagnosis in the clinical domain. Poor definition of the clinical added value, emphasis on statistical association in the absence of careful statistical calculations to justify sample sizes needed among others, alongside poor understanding of the influence of lifestyle and diet on cancer lipid profiles constitute important areas of improvement in future data modeling. We hope that our recommendations will save time and effort in the early evaluation of promising leads towards successful translation in a manner that reduces the discordance between initial phase promise and late-stage performance. It must be emphasized that the recommendations provided in this manuscript aim to enhance untargeted ambient MS methods that do not utilize pre-separation of analytes (as done in conventional lipidomics), such as those obtained with hand-held MS probes. As of late, ion mobility separation is being incorporated into many MS sources, which will undoubtedly enhance the robustness of ambient MS datasets. Therefore, appropriate revisions to these guidelines are envisioned as the field of ambient MS evolves to incorporate new separation technologies. 

## Figures and Tables

**Table 1:** Challenges and sources of potential failures in conventional targeted biomarker discovery efforts. This table lists current challenges in different phases of biomarker discovery. Parallels, at each phase of discovery, validation, translation, evaluation, and implementation can be drawn to be used as guidelines for potential pitfalls of ambient mass spectrometry profiling. Reproduced with permission [55].

	Current Problems	Potential Solutions
Discovery	Poor design, conduct, and analysis	Methodological rigor
	Unaccounted multiplicity	Appropriate use of statistics
	Small studies	Larger, collaborative studies
	Extreme case selection	Proper case-control or cohort selection
	Nonrigorous exploratory nature of studies	More rigorous training of scientists
	Poor reporting	Use of reporting standards
	Selective reporting	Preregistration
	Spin in interpretation	Careful editorial and peer-review
Validation	Any and all problems seen in discovery studies	Similar solutions, as above
	Lack of replication efforts	Incentives for running replication studies
	Inbred replications (same populations, same investigators)	More emphasis on external, independent validation
	Incomplete, suboptimal validation	Careful consideration of independence
	No systematic reviews	Good-quality systematic reviews
	Inflation in early, small studies	Large validation studies, ideally from collaborations without bias
	Spurious variability in measurements, methods, analyses across studies	Standardization and harmonization of processes, collaborative consortia
Transition to clinical translation	Inappropriate perusal of clinical translation	Rigorous systematic reviews
	Poor prioritization	Rigorous umbrella reviews
	Sponsor bias driving translation urge	Independent assessment of the evidence
	Inappropriate stagnation without clinical translation	Incentives to translate
Evaluation	Focus only on accuracy and process measures	Emphasize patient outcomes
	Few randomized trials of biomarkers	Promote randomized trials of biomarker use
	Use for unclear informational purposes	Evaluate utility of information for the sake of information and potential collateral harms
	Improper use for selection and stratified/subgroup analyses in trials	Validation of utility of stratified/subgroup analyses
Implementation and deimplementation	Poor understanding of the use of biomarkers in real-life settings	Implementation studies assessing use and outcomes in diverse settings
	Lack of rigorous guidelines	Standardized, nonconflicted guidelines
	Discordant guidelines	Strengthening of regulation for biomarkers
	Not well-defined regulatory landscape	Testing of utility of long-used biomarkers
	Entrenched useless biomarkers	Overcoming resistance from conflicted stakeholders, higher barrier for reimbursement
	Resistance to deimplementation even with convincing negative evidence	

## Data Availability

The data presented in this study (Figure 3A, re-analyzed previously published results [9]) are available on request from the corresponding author. The raw data is not publicly available due to Ethical research guidelines that prohibit sharing and transfer of anonymized human specimen raw data without institutional authorization.
